# Challenges in lentiviral vector production: retro-transduction of producer cell lines

**DOI:** 10.3389/fbioe.2025.1569298

**Published:** 2025-05-29

**Authors:** Maximilian Klimpel, Christin Obert, Monica Terrao, Parameswari Singh, Haiyan Wei, Chao-Guang Chen, Andreas Gille, Silke Wissing, Holger Laux

**Affiliations:** ^1^ CSL Innovation GmbH, Marburg, Germany; ^2^ CSL Innovation, Melbourne, Australia; ^3^ CSL Behring, Pasadena, CA, United States

**Keywords:** lentiviral vector (LV), viral vector production, retro-transduction, auto-transduction, cell and gene therapy (CGT), stable producer cell line, LDL-R knockout, VSV-G (vesicular stomatitis virus G glycoprotein) pseudotyped HIV-1 derived vector particles

## Abstract

The increasing demand for lentiviral vectors (LVs) has led to the development of several stable cell lines and production methods over the last 2 decades in order to increase titers and yields, reduce production costs and improve the safety of the vector product. However, the phenomenon of retro-transduction, which describes the transduction of LV producer cells by self-produced LVs, remains largely unaddressed in the context of LV production. Recent research has focused on various approaches to reduce the impact of retro-transduction on LV yield and process performance. This article reviews existing and new research data that highlights the impact of retro-transduction in LV manufacturing. In addition, a perspective on current advances to reduce retro-transduction is provided and a potential novel strategy called ENV-Y is presented, which could not only reduce the impact of retro-transduction but also facilitate the subsequent LV downstream process.

## 1 Introduction

Lentiviral vectors (LVs) are an important tool for cell and gene therapies due to their ability to stably integrate their genome into dividing and non-dividing cells (Kotterman et al., 2015). However, the production of high-quality LVs in large quantities is hampered by different upstream and downstream processing challenges, such as only moderately achievable infectious titers and low LV stability (Merten et al., 2016). A further issue that has not been sufficiently addressed in the field of viral vector production is the transduction of virus-producing cells by the virus that has been expressed by those cells. The phenomenon is caused by the lack of superinfection interference in retroviral vector-producing cells and is also referred to as retro-transduction, auto-transduction or self-transduction (Banos-Mateos et al., 2024; Elizalde and Ramírez, 2021; Klimpel et al., 2023; Schaser et al., 2021; Vogt et al., 2001; Williams-Fegredo et al., 2024). The most widely used envelope for LV-pseudotyping is the vesicular stomatitis virus glycoprotein G (VSV-G), which targets the highly ubiquitous expressed low-density lipoprotein receptor (LDLR) and related family members for cellular binding and uptake (Finkelshtein et al., 2013). The ubiquitous expression of the LDLR explains the broad tropism of VSV-G for therapeutically relevant cells. However, its expression on HEK293(T) cells also mediates the retro-transduction of virus-producing cells. The phenomenon reduces the number of harvestable LVs and decreases the overall LV yield obtained per run (Elizalde and Ramírez, 2021; Schaser et al., 2021). Another effect is the accumulation of integrated vector genomes and increasing transgene expression in producer cells, which may impact cell growth, viability and productivity. That may result in increased levels of host cell DNA and proteins, which need to be reduced in the final LV product. Retro-transduction may also negatively affect the particle to infectivity ratio as only infectious LVs are lost. It can also be hypothesized that those LVs exhibiting the highest infectivity are most likely to be lost by retro-transduction of producer cells, resulting in a bias towards high VSV-G LV depletion.

Recently, several strategies have been described to reduce retro-transduction during LV production. One strategy is to knock down or knock out the LDLR gene or genes relevant for the LDLR function in LV-producing cell lines with the aim of reducing or abolishing its expression. However, although this strategy has been reported to increase LV yield and quality by some groups, other groups have observed no benefit, but an impairment of cellular functions related to lipid metabolism (Banos-Mateos et al., 2024; Han et al., 2021; Otahal et al., 2015; Schaser et al., 2021). While great advances have been made in the field of viral vector production, the impact of retro-transduction in production processes is often neglected or not addressed. In this perspective article, we characterize the extent and impact of retro-transduction for stable inducible producer cell clones and pools based on the stable packaging cell lines GPRG and GPRTG, which have been used to produce different LVs in adherent or suspension cultivation mode (Bonner et al., 2015; Choi et al., 2015; Greene et al., 2012; Klimpel et al., 2023; Klimpel et al., 2024; Klimpel et al., 2025; Throm et al., 2009; Wielgosz et al., 2015). LV productions using adherent producer pools were performed in cell stacks and LV productions using suspension producer clones were performed in stirred-tank bioreactors. We found a surprisingly high number of integrated vector genomes in both expression systems. Based on the findings, potential strategies for reducing retro-transduction are reviewed as the *status quo* and critically discussed, along with our newly generated research data. Finally, a future perspective on the implications for LV production processes is given.

## 2 The degree of retro-transduction in LV producer cells and impact on the vector product

The lack of superinfection interference in retroviral vector producer cells, specifically by vectors pseudotyped with VSV-G, has been described a few years after the establishment of stable inducible packaging cell lines for the expression of those vectors (Vogt et al., 2001). A major problem associated with retro-transduction of producer cells is the reduced number of infectious harvestable vector, which increased the production costs per LV unit. The loss of functional LV has been assessed and estimated to be in a range of 60%–90% (Banos-Mateos et al., 2024; Elizalde and Ramírez, 2021; Williams-Fegredo et al., 2024). However, these investigations of retro-transduction in LV production have been conducted for processes that rely on transient transfection of vector components.

**FIGURE 1:**
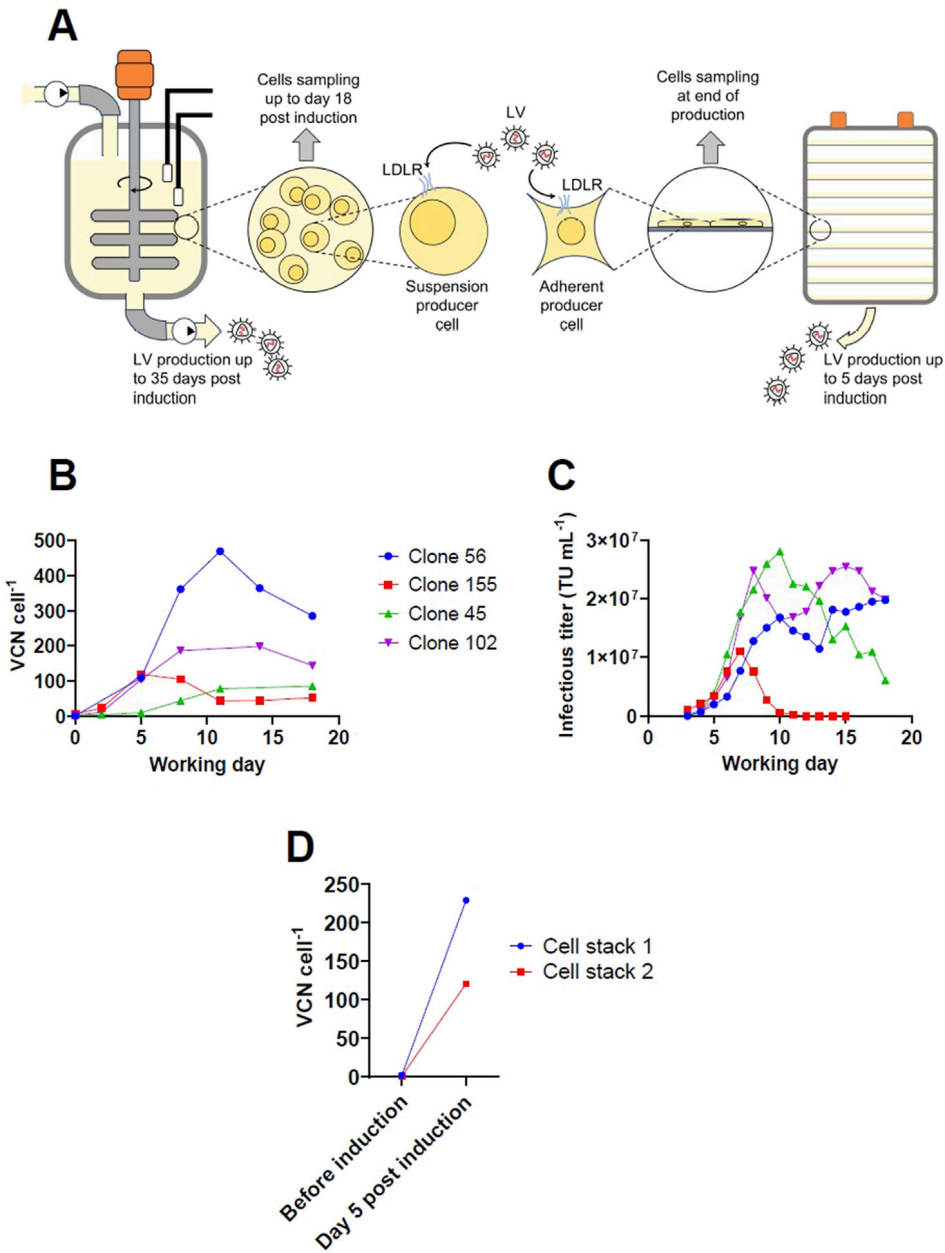
Investigation of retro-transduction for stable inducible adherent and suspension producer lines expressing a WAS-T2A-GFP LV upon induction. **(A)** Schematic drawing of the experimental set-up for LV production and mechanism of retro-transduction. LV production using suspension cells was performed in 5 L stirred-tank bioreactors using stable producer clones based on the stable inducible packaging cell lines GPRGs and GPRTGs. LV production using adherent cells was performed in 10-layered cell stacks using a stable producer pool based on the stable packaging cell line GPRTG. **(B)** Integrated LV genomic vector copy number determined by digital droplet polymerase chain reaction for different stable GPRGs and GPRTGs producer clones over the production period in 5 L stirred-tank bioreactors. **(C)** Corresponding infectious titers for LV production in 5 L stirred-tank bioreactor determined in the collected harvest. **(D)** Integrated LV genomic vector copy number determined by digital droplet polymerase chain reaction for a stable GPRTG producer pool before induction (time point of seeding) and at end of production (5 days post induction).

Our group has previously described a LV production process using Tet-off inducible suspension cell lines based on the packaging cell lines GPRG and GPRTG (Klimpel et al., 2023; Klimpel et al., 2024). For the bioreactor production campaign that was previously published in Klimpel et al. (2024), the retro-transduction rate was determined at different time points of the process up to day 18 post induction (Figure 1A). For LV productions using an adherent GPRTG producer pool, the retro-transduction rate was determined by collecting cells at the end of the production (Figure 1A). To assess the retro-transduction rate, the integrated LV genome copy number in the producer cells was determined by digital droplet PCR after extracting cellular genomic DNA. As stable producer cells were used in this study, different primer sets were used to differentiate between the vector cassettes that were previously integrated by stable transfection and the vector copy number (VCN) of LV genomes integrated by retro-transduction of the producer cells. One primer set was designed to target the WPRE sequence located on the LV genome. In addition, multiple primer sets were designed, targeting the backbone of the vector cassette that is stably integrated in the producer cells, to ensure that those vector copies are not considered for the retro-transduction determination. The kinetic and maximum VCN of integrated LV genomes in suspension cells was found to be variable between producer clones. The highest value of 469 VCN cell^-1^ was determined for clone 56 at working day 11 (Figure 1B). Interestingly, when the results of different producer clones are compared, no correlation was found between the peak or average infectious titer determined in the supernatant and the integrated VCN (Figures 1B,C) (correlation matrix performed with GraphPadPrism 10, both p values > 0.85, not displayed). The degree of retro-transduction has been also assessed for an adherent GPRTG producer pool. After a 5-day production in cell stacks, a value of up to 229 VCN cell^-1^ was determined (Figure 1D). Based on the total virus yield obtained and the maximum cell density reached in both the adherent- and suspension-based expression system, it can be estimated that 87%–97% of infectious virus is lost by retro-transduction, highlighting the problem of retro-transduction in LV manufacturing. In contrast to the adherent LV expression system, where cells can only be sampled at the end of production, LV production in suspension bioreactors allows to determine the VCN at different times during the production process. For all the clones, the VCN initially showed a gradual increase. In the further course, clone 45 reached a plateau, while the VCN for clone 56, 102 and 155 decreased after reaching a maximum at individual time points. It is possible that several days after LV transduction, genomic DNA vector copies are still detected that are not stably integrated into the cell’s genome (Scaramuzza et al., 2013). That may explain the trend for clone 155, for which the VCN was stabilizing at working day 12 at approximately 50 VCN cell^-1^ after the expression of infectious vector dropped below the detectable limit. However, the VCN for clone 56 also decreased after reaching a peak, although the determined infectious titers remained >1 × 10^7^ TU mL^-1^. The data indicate that the events of retro-transduction decrease over the production time and a potential cellular resistance is developed, or that individual producer cells are proliferating at different growth rates, as the determined VCN is only an average of the whole cell population. Cells that have a higher VCN likely express higher levels of the transgene, which may reduce the growth rate compared to cells with lower transgene expression levels and can result in selective growth. The increasing VCN could result in genomic instability, disruption and dysregulation of cellular genes, or in a potential depletion of transcription factors recruited to the transgene. We observed a gradual reduction of the growth rate over the production time, indicating an impact of the increasing VCN on cellular functions. However, further investigations are required, as other factors like the persistent expression of toxic vector components like VSV-G may also affect cell growth.

More recently, some groups have published concepts to reduce retro-transduction in LV manufacturing and the associated adverse effects on process performance and vector quality. In the following, different approaches for reducing retro-transduction during LV production are reviewed and critically discussed. In addition, other novel potential approaches are discussed, and a future perspective for LV manufacturing is given.

## 3 Strategies for reducing retro-transduction

### 3.1 A strategy with controversial findings: Generation of LDLR knockout cell lines to reduce retro-transduction

The discovery of the LDL receptor and its family members as a major cellular entry port of the vesicular stomatitis virus (Finkelshtein et al., 2013) provided the basis for the concept of preventing retro-transduction on producer cells for VSV-G pseudotyped LVs by reducing or abolishing LDLR expression (Schaser et al., 2021). The interaction between VSV-G and LDLR is specifically mediated by two distinct cysteine-rich domains of LDLR, called CR2 and CR3, and two basic residues on the glycoprotein (Nikolic et al., 2018).

Schaser et al. (2021) have generated LDLR negative packaging cell lines based on HEK293T cells growing in suspension by a LDLR gene knockout, without further specifying the used knockout strategy. The authors have investigated LV productivity and LV retro-transduction rate upon transient transfection of plasmids carrying the required vector components in these cell lines. It was shown that the retro-transduction of the virus producing cells is up to 45% lower compared to the wild-type HEK293T cells, which increased the number of harvestable LVs and consequently the infectious titer. Reduced retro-transduction was quantified by reduced mean fluorescence intensity levels when a GFP vector was expressed and by reduced integrated LV genome copy numbers in the producer cells. In addition, the authors found that VSV-G expression resulted in reduced toxicity to the producer cells, as evidenced by increased cell viability. As the fusion activity of VSV-G is largely determined by pH and occurs predominantly under acidic conditions, which can occur in cultivation methods such as cell culture flasks without pH control, the reduced toxicity can be explained by reduced syncytia formation (Beilstein et al., 2020; Ferlin et al., 2014). It may be hypothesized that syncytia formation is enhanced by interference of the ligand binding domain of LDLR and VSV-G between neighboring cells that are expressing both the receptor and the envelope. The authors demonstrated stable expression of VSV-G in the LDLR knockout cell lines. In addition, LDLR negative clones stably expressing VSV-G were selected, that exhibit superior LV productivity upon transient transfection of the remaining vector components (Schaser et al., 2021).

The group from Han et al. (2021) has investigated the impact of multiple CRISPR-Cas9-mediated gene knockouts on transient LV production, including the knockout of the LDLR gene. The knockout of the LDLR gene was analyzed by sequencing and ICE Synthego analysis and showed an indel percentage and a knockout score of 93% in both cases. The lack of LDLR expression was also verified by flow cytometry. The authors reported a twofold increase in viral titer when only the LDLR gene was knocked out. However, in contrast to the findings from Schaser et al. (2021), Han et al. (2021) found that LDLR-negative and the parental HEK293T cells were equally transduced by the produced virus. The authors concluded that the increased titer formation is likely due to a reduced formation of LDLR-VSV-G complexes in the ER-Golgi intermediate compartment and subsequent degradation in aggresome/autophagosome, which has been previously hypothesized by another group (Otahal et al., 2015).

While Han et al. (2021) and Schaser et al. (2021) reported a positive impact of the LDLR knockout on vector titer formation, Banos-Mateos et al. (2024) have recently reported contradictory findings. The authors performed a LDLR gene knockout on the producer cells using the CRISPR-Cas9 technology with two single guide RNAs targeting exon two and exon six to abolish LDLR expression. Clones carrying the gene deletion were selected by PCR and the lack of LDLR expression was verified by flow cytometry. The selected LDLR knockout cell clones were transiently transfected with plasmids carrying the required vector components for LV production. The integrated VCN per producer cell at the end of production was significantly reduced. However, the infectious and physical titers obtained were not significantly higher or even reduced, depending on the producer clone. The authors hypothesized a negative impact of the abolished LDLR expression on the cellular cholesterol levels, which in consequence may impact LV infectivity and stability. The authors reported that lower cholesterol levels were found in the investigated knockout cell line compared to the parental HEK293T cell line. Additional cholesterol supplementation did not restore cellular cholesterol levels for the knockout cell line.

Another published work investigated a CRISPR-Cas9-mediated knockout of the LDLR gene in HEK293T cells to prevent retro-transduction (Tijani, 2018). The LDLR knockout HEK293T cells were incubated with LVs pseudotyped with different glycoproteins including VSV-G to investigate its transducibility. The author reported only a modest reduction of LV transduction in the knockout cell line. However, it needs to be considered that some remaining LDLR expression was found in the knockout cells and no clonal selection step has been performed, indicating that a screening for suitable knockout clones is required to benefit from an LDLR knockout (Tijani, 2018).

**FIGURE 2:**
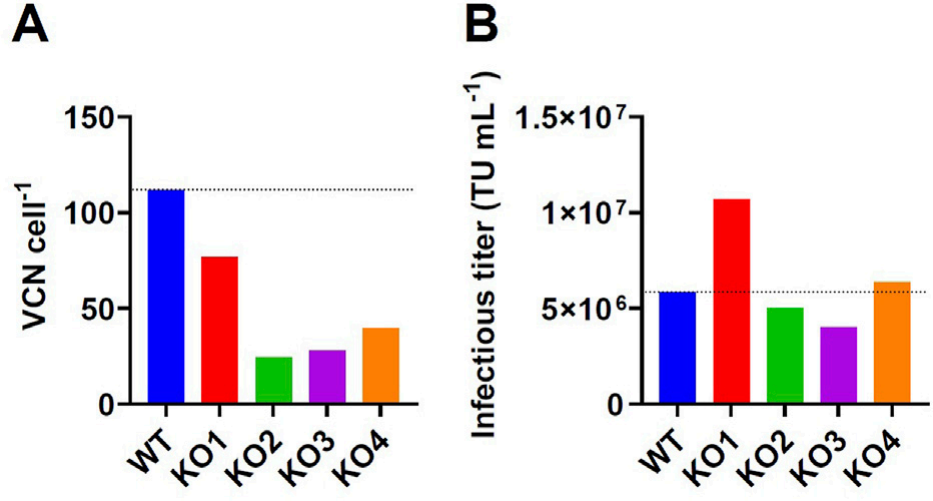
Results of CRISPR-Cas9-mediated LDLR gene knockout in a stable GPRG-GFP producer clone. The wildtype cell clone (WT) and selected LDLR knockout clones (KO 1-4) were seeded in T75-flasks and induced by doxycycline removal. **(B)** Three consecutive harvests were collected and pooled for determination of infectious titer on HEK293T cells. **(A)** Producer cells at the end of production were collected to determine the integrated LV genomic vector copy number by digital droplet polymerase chain reaction as an indicator for retro-transduction.

Previously published work investigated the effect of an LDLR gene knockout on transient LV production. Since our group observed high integrated VCN for different stable GPR(T)G producer cells after induction of LV production (Figures 1B,D), we have investigated the effect of a CRISPR-Cas9-mediated LDLR gene knockout on retro-transduction and infectious titer formation using an adherent stable GPRG-GFP producer clone. Four LDLR negative clones and the wildtype clone were compared for LV production in semi-perfusion mode and cells were harvested at the end of production to determine retro-transduction. Retro-transduction was reduced for all four LDLR knockout clones at the end of production, showing values of 24–77 VCN cell^-1^ compared to 112 VCN cell^-1^ for the wildtype clone (Figure 2A). However, only clone KO1 showed a noticeably higher infectious titer of 1.07 × 10^7^ TU mL^-1^ compared to 5.85 × 10^6^ TU mL^-1^ produced with the wildtype clone (Figure 2B), although the reduction in retro-transduction was the lowest for KO1 (Figure 2A). The remaining clones produced a similar or lower infectious titer compared to the wildtype clone. The data suggest that the LDLR knockout can reduce retro-transduction and may be able to increase infectious titer formation even in stable cell lines. However, LDLR knockout may also have a negative effect on LV productivity, which can be explained by an impairment of lipid metabolism as described by Banos-Mateos et al. (2024). It should also be noted that potential off-target effects that may result from CRISPR/cas9 knockout have not been investigated (Guo et al., 2023; Zhang et al., 2015).

Overall, available data show that the LDLR gene knockout approach can increase LV yields and reduce retro-transduction in producer cells, but the approach does not seem to be successful in all instances and requires an investigation for individual cell clones and expression systems. As LDLR plays an important role in cholesterol and lipid metabolism, knockout of the gene may lead to changes in the cell membrane, which in turn may affect viral titer, infectivity and stability of the LVs produced. It is also important to note that LDLR gene knockout only reduces VSV-G-mediated transduction, but does not completely abolish it, as other LDL-R family members serve as alternative cellular entry points (Finkelshtein et al., 2013; Nikolic et al., 2018). In addition, the widely used CRISPR-Cas9 technology harbors the risk of off-target effects, which could lead to unwanted or adverse effects to the cellular genome (Guo et al., 2023; Zhang et al., 2015).

### 3.2 Alternative approaches to reduce retro-transduction

Next to the LDLR gene knockout, other strategies to reduce the LV retro-transduction during transient LV production were investigated by Banos-Mateos et al. (2024). First, the authors showed that infection of VSV-G pseudotyped LVs is inhibited by addition of anti-VSV-G antibody. On the same note, the authors showed that non-enveloped produced vectors do not infect HEK293T cells and that the integrated VCN determined for producer cells to produce non-enveloped vectors are significantly lower compared to those to produce VSV-G pseudotyped LVs. Based on these findings, producer cells were generated that overexpress soluble LDLR, which interacts with the LVs VSV-G envelope to prevent the cellular internalization. While the degree of retro-transduction could be reduced, indicated by a 40% lower VCN compared to the control, the approach did not result in increased infectious or physical LV titers. Similar results were obtained when the functional LDLR domain, called cysteine-rich domain 3 (CR3), was added as a peptide to the cell culture medium after transfection. It also needs to be considered that the addition of molecules preventing LV transduction requires a strategy for the release and reconstitution in a subsequent downstream process.

Another approach investigated by Banos-Mateos et al. (2024) aimed to block receptors for internalization of VSV-G pseudotyped LVs by co-expression of soluble receptor-associated protein (RAP), a molecular chaperon for LDLR-related family members (Willnow et al., 1995; Willnow et al., 1996). Some cellular *in-vitro* models showed that exogenous RAP blocks ligand binding to all LDLR related family members, except for LDLR itself (Finkelshtein et al., 2013; Nikolic et al., 2018). However, Banos-Mateos et al. (2024) also assume an interaction between LDLR and RAP based on a previously identified crystal structure (Fisher et al., 2006), which is also supported by findings of another group (Kurasawa et al., 2013; Marakasova et al., 2021). Banos-Mateos et al. designed different RAP constructs to improve its secretion and stability. Although the determined integrated VCN per producer cell was reduced by co-expression of all RAP constructs, some constructs also reduced LV productivity, which may be due to interference with the transport of LDLR related family members and a resulting change in the cellular metabolism, as hypothesized by the authors. Only one RAP construct was able to reduce the VCN per producer cell by 40% with an average increase in infectious titer of 36% compared to the control (Banos-Mateos et al., 2024).

A simpler approach to reduce LV retro-transduction was recently published by Williams-Fegredo et al. (2024). The authors demonstrated that lowering the pH after transient transfection of HEK293T-cells for the production of VSV-G pseudotyped LVs significantly reduces the degree of retro-transduction. A pH shift to 6.7-6.8 post-transfection resulted in a sevenfold reduction of the integrated VCN at the end of the production process compared to an operation at pH 7.0 (Williams-Fegredo et al., 2024). The mechanism is based on the fact that VSV-G can undergo a pH-dependent, reversible conformational change between a pre-fusion and a post-fusion state (Beilstein e al., 2020). The LDLR-binding domains only bind VSV-G in its pre-fusion conformation, which is present at neutral or high pH, while no interaction occurs in its post-fusion conformation, which is present at acidic pH (Nikolic et al., 2018). The positive effect of lowering the pH during LV production to increase the functional yield has been previously described by other groups (Holic and Fenard, 2016; Powers et al., 2020; Stibbs et al., 2024; Valkama et al., 2017), which may be at least partially due to the same mechanism. In contrast, although Williams-Fegredo et al. (2024) successfully reduced retro-transduction of producer cells during LV production, the peak infectious titers obtained were comparable across the different pH set points studied and peak titers were obtained even earlier at higher pH, which did not allow to benefit from increased functional yield in production in batch mode. However, the authors found that the peak infectious titer was maintained for a prolonged time at lower pH compared to a production at higher pH, suggesting that a continuous harvesting strategy in combination with a low pH set point may allow to benefit from increased LV yields (Williams-Fegredo et al., 2024). A clear benefit of the presented strategy is that it is easy to implement, scalable and most likely widely applicable for the production of VSV-G pseudotyped LVs.

Another approach is to engineer LVs for more specific targeted transduction, which can reduce or prevent the retro-transduction of producer cells while increasing the transduction efficiency of target cells. This can be achieved by using alternative envelopes that have distinct cell entry mechanisms and target receptors expressed on the target cells but not, or only weakly, on producer cells. Various glycoproteins derived from different viruses have been used, such as those from the Measles virus (Funke et al., 2009; Humbert et al., 2012), Baboon endogenous virus (Costa et al., 2016; Girard-Gagnepain et al., 2014), Sindbis virus (Morizono et al., 2010), Rabies virus (Hislop et al., 2014), Nipah virus (Palomares et al., 2012), and Lymphocytic Choriomeningitis Virus (Stein et al., 2005). Although retro-transduction of producer cells has not been investigated yet, the use of alternative envelopes with a narrow cell tropism presents an appealing approach compared to the VSV-G glycoprotein. A drawback of using alternative LV envelopes is the necessity for comprehensive new safety evaluations and tests including vector characterization, tropism testing, immunogenicity assessments, and toxicology studies.

Another strategy involves the simultaneous incorporation or fusion of antibodies or antibody fragments and a fusogenic protein onto the lentiviral surface to enhance specificity for particular cell types (Berckmueller et al., 2023; Yang et al., 2008; Yang et al., 2006). The incorporation of an antibody or antibody fragment ensures LV binding specificity for target cell types, making this approach attractive for *in-vivo* use. The fusogenic protein is engineered to abolish its specific receptor binding properties while retaining its membrane fusion activity (Berckmueller et al., 2023; Yang et al., 2008; Yang et al., 2006).

An alternative method involves the subsequent modification of produced LVs with cell-targeting antibodies. Viral vectors can be produced that display antibody-binding domains, enabling the subsequent conjugation of cell-targeting antibodies (Ohno et al., 1997; Wu et al., 2012). The use of bispecific antibodies, as described by Parker et al. (2020), allows these antibodies to bind both the produced LVs and the receptors of target cells. A major advantage of this method is that the same produced LV can be modified to target several different cell types. However, this method requires the separate engineering and production of antibodies at clinical grade, making the overall process more complex and cost-intensive. In addition, several other factors must be considered like impact on fusogenic properties, vector stability, vector titer and limitations through immunogenicity induced by antibodies or antibody fragments.

### 3.3 ENV-Y: a novel technology to reduce retro-transduction in VSV-G pseudotyped LV production

Our group has developed novel recombinant VSV-G-binding proteins called ENV-Y (Gille and Fabri, 2024). This type of molecule not only addresses the challenges of retro-transduction of producer cells and syncytia formation induced by the fusion activity of VSV-G, but also provides an approach for a highly selective LV purification method. ENV-Y is a fusion protein of the fragment crystallizable antibody region (Fc region) with the VSV-G binding domain of LDLR, which can bind and neutralize lentiviral VSV-G, thereby preventing LV transduction of producer cells (Supplementary Figure S1A). Depending on the pH during LV production, binding of ENV-Y to VSV-G expressed on the cells surface may reduce syncytia formation induced by the fusion activity of VSV-G, and thereby reducing cellular toxicity (Beilstein et al., 2020). Different ENV-Y molecules have been expressed, varying in number of C2 and C3 domains and linkers, and purified for preliminary experiments. Surface plasmon resonance experiments showed that ENV-Y binds to VSV-G with high affinity, and LV neutralization was demonstrated by dose-dependent reduction of transduction in HEK239T cells (data not shown). The Fc region of the ENV-Y molecule enables selective purification using a protein A ligand. After capturing LVs coated with ENV-Y using a protein A ligand, LVs may be eluted by using a calcium-free EDTA containing buffer at low pH. As the binding of LDLR and VSV-G is reversible, a pH of <6.2 changes reversibly from its pre-to a post-fusion conformation, which reverses the bonding of ENV-Y and VSV-G. Rapid dissociation of VSV-G from ENV-Y molecules at pH 6 has been demonstrated by surface plasmon resonance experiments (Supplementary Figure S1B). The addition of EDTA as a chelator for divalent cations should further promote the release of LVs from ENV-Y, as the interaction between VSV-G and LDLR is calcium depended (Finkelshtein et al., 2013). Preliminary data generated indicate that the ENV-Y molecule has the potential to increase yield and quality in LV manufacturing by decreasing retro-transduction and enabling affinity purification and release using mild elution conditions. Further development work is required to investigate and implement its full potential, including the development of LV producer cells stably expressing ENV-Y.

## 4 Discussion and outlook

Considering the number of production processes and producer cell lines that have been developed, retro-transduction remains a neglected and poorly understood phenomenon. Recent reports have highlighted the need for further investigation of retro-transduction, leading to the development and implementation of various strategies aiming to prevent it. Although several approaches result in reduced retro-transduction of LV-producing cells, in many cases no or only a moderate positive effect on infectious titer formation has been achieved. The use of alternative envelopes presents a promising approach to limit tropism exclusively to target cells, thereby reducing retro-transduction of producer cells. However, further investigations are required to ensure the safety of these novel vectors. Despite these advancements, LVs pseudotyped with the well-studied VSV-G glycoprotein remain the most widely used LVs in clinical applications.

A strategy for producing VSV-G pseudotyped LVs that has shown a positive impact on infectious titer formation and retro-transduction in some cases is the LDLR knockout, although LDLR related family members can serve as an alternative LV entry port (Finkelshtein et al., 2013; Nikolic et al., 2018). A major drawback is that this strategy requires a cumbersome cell line development process to select clones that are LDLR negative but retain high LV productivity and appropriate cell growth. In addition, since LDLR gene knockout can affect cellular lipid metabolism, the potency of the produced LVs needs to be assessed, ideally in the therapeutically relevant cells, as surrogate cells such as HEK293T cells are readily transducible. Available data indicate that the strategy of LDLR gene knockout for reduced retro-transduction may be specific for each expression system and requires an individual investigation. Similar conclusions can be drawn from the strategy of overexpressing RAP constructs to block LDLR family related receptors, which can also negatively impact LV productivity. Approaches that make use of more universal mechanisms which reduce the risk of interference with the cellular metabolism are desired to enable an application for various existing cell lines and production processes. Theoretically, proteins that bind to VSV-G to neutralize LVs and prevent its interaction with receptors on the cells surface should be universally applicable, such as the soluble LDL. However, these approaches create the need to develop a process to remove blocking agents and recover LVs in its functional form. Our presented ENV-Y concept may be suitable to prevent retro-transduction and enables the use of a widely used mechanism for protein purification to enable gentle LV recovery and a selective purification. To make the ENV-Y approach cost-effective in perfusion processes, the molecule needs to be stably expressed in the producer cells. Therefore, similar like for the LDLR knockout approach, a cell line development process with clone selection is required. In contrast, the recently published mechanism of reducing retro-transduction by lowering the pH is easy and fast to implement for different cell lines and production processes (Williams-Fegredo et al., 2024), providing a clear benefit. However, cellular productivity at reduced pH seemed to be lower at earlier process time points. Peak functional LV titers were only maintained for a longer period but not higher compared to the control process, indicating that a perfusion process would be required to benefit from increased LV yields (Williams-Fegredo et al., 2024).

LV production in perfusion mode has been described to increase functional vector yields compared to production in batch mode (Ansorge et al., 2009; Klimpel et al., 2023; Manceur et al., 2017; Tona et al., 2023; Tran and Kamen, 2022). Despite achieving higher vector titers by increasing biomass concentration, temperature-dependent LV inactivation (Ansorge et al., 2010; Higashikawa and Chang, 2001; Klimpel et al., 2025) and retro-transduction of producer cells may be reduced. It can be hypothesized that the degree of retro-transduction is mainly determined by the LV titer and the residence time in the bioreactor. In batch mode, the produced vector remains in the cell culture for the entire process duration, while in perfusion mode, the vector is continuously removed from the bioreactor, potentially reducing retro-transduction. An even lower retro-transduction may hypothetically be achieved by increasing perfusion rates, thereby reducing the vector’s residence time and transduction events of producer cells. However, this approach may lead to lower LV titers due to dilution, significantly increasing medium costs and necessitating adaptations in the downstream process to handle increased harvest volumes. Further research is required to understand the specific effect of perfusion processing and medium exchange rates on retro-transduction, as other mechanisms such as reduced temperature-dependent LV inactivation and the removal of cellular metabolites can affect vector yields.

An alternative approach to reduce retro-transduction may be a screening for small molecules that interfere with the specific binding between VSV-G and cellular receptors for LV internalization, as the structural basis for the recognition of LDLR family members by VSV-G is described and understood (Nikolic et al., 2018). The desired molecule is ideally in-expensive, non-toxic to the cell culture and can be easily removed during downstream processing. The approach would enable an easy implementation for various production processes and ideally does not interfere with the cellular lipid metabolism, which is crucial for the production of infectious LVs.

In summary, the influence of retro-transduction on LV production has recently received increasing attention. A better understanding of the mechanisms involved has led to the development of various techniques to reduce retro-transduction. However, the methods investigated often only allow a reduction in retro-transduction. In addition, some approaches are difficult to implement and may also have a negative impact on productivity. It is also worth mentioning that the majority of existing research investigated retro-transduction in transient production processes, although there is an increased interest for stable packaging and producer cell lines. Further research is needed to better characterize and control the phenomenon of retro-transduction and benefit from improved LV yields.

## Data Availability

The original contributions presented in the study are included in the article/Supplementary Material, further inquiries can be directed to the corresponding author.

## Supplementary material

SUPPLEMENTARY FIGURE S1
**(A)** The concept of ENV-Y as a novel molecule to prevent retro-transduction of LV producer cells and facilitate the subsequent downstream process. ENV-Y is binding to produced VSV-G pseudotyped LVs to prevent retro-transduction of producer cells. The Fc domain of the ENV-Y molecule is used to capture ENV-Y coated LVs using a protein A ligand. LVs are eluted using a buffer containing EDTA at pH 6. **(B)** Surface plasmon resonance experiments showing pH-dependent interaction of ENV-Y and VSV-G. VSV-G is rapidly dissociating from ENV-Y molecules at pH 6.
