# Supplementary figures and images for: Challenges in lentiviral vector production: retro-transduction of producer cell lines
Source: Front Bioeng Biotechnol. 2025 May 29;13:1569298. doi: 10.3389/fbioe.2025.1569298 (PMC12159608; doi:10.3389/fbioe.2025.1569298)

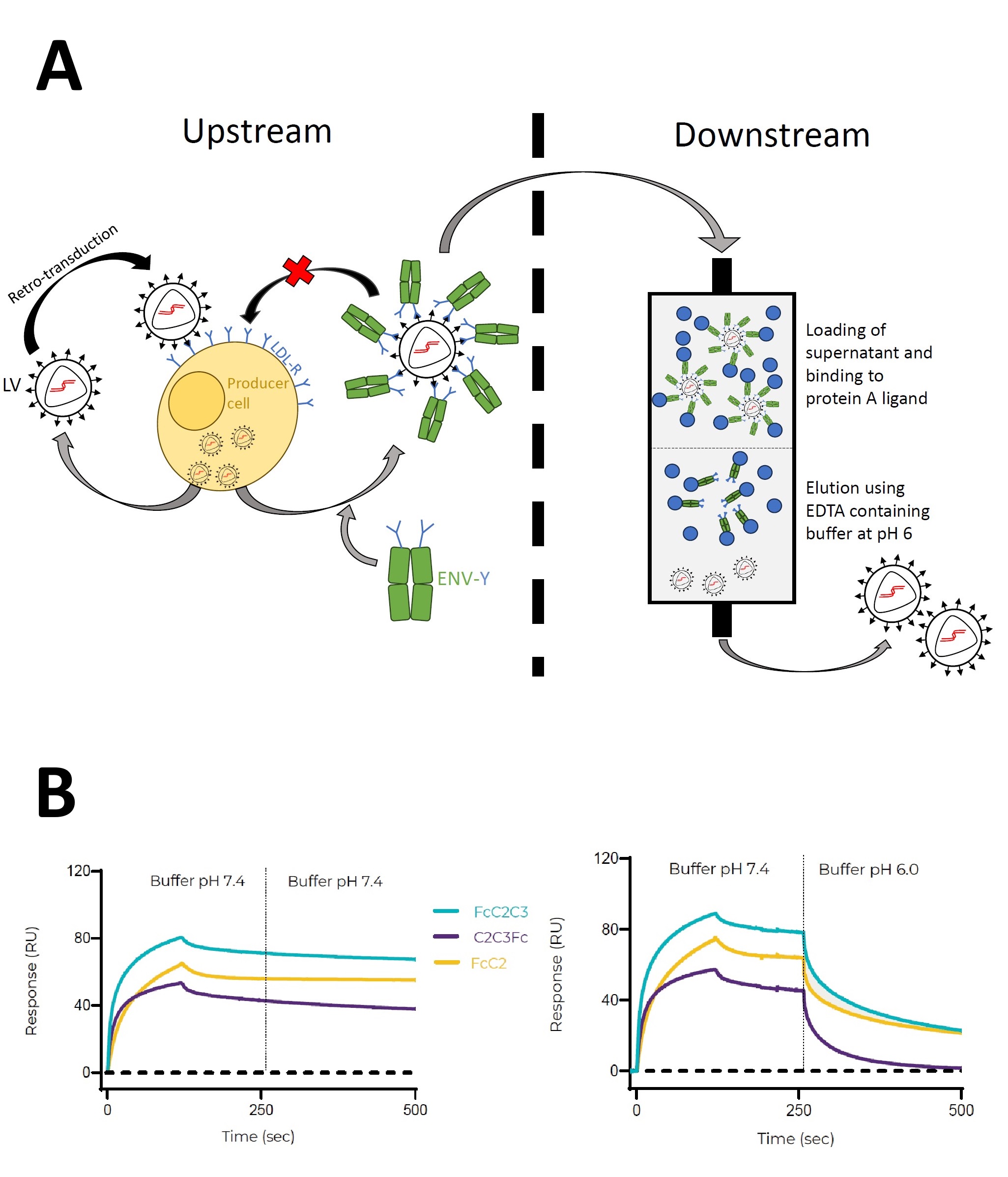

Supplement: Supplementary file 1 [file Image1.jpg]
